# Rescue of Photoreceptor Degeneration by Curcumin in Transgenic Rats with P23H Rhodopsin Mutation

**DOI:** 10.1371/journal.pone.0021193

**Published:** 2011-06-29

**Authors:** Vidyullatha Vasireddy, Venkata R. M. Chavali, Victory T. Joseph, Rajendra Kadam, Jonathan H. Lin, Jeffrey A. Jamison, Uday B. Kompella, Geereddy Bhanuprakash Reddy, Radha Ayyagari

**Affiliations:** 1 Jacobs Retina Center, Department of Ophthalmology, University of California San Diego, La Jolla, California, United States of America; 2 Department of Pathology, University of California San Diego, La Jolla, California, United States of America; 3 Department of Pharmaceutical Sciences, University of Colorado Denver, Aurora, Colorado, United States of America; 4 Opthy-DS Inc., Mattawan, Michigan, United States of America; 5 National Institute of Nutrition, Hyderabad, India; Center for Regenerative Therapies Dresden, Germany

## Abstract

The P23H mutation in the rhodopsin gene causes rhodopsin misfolding, altered trafficking and formation of insoluble aggregates leading to photoreceptor degeneration and autosomal dominant retinitis pigmentosa (RP). There are no effective therapies to treat this condition. Compounds that enhance dissociation of protein aggregates may be of value in developing new treatments for such diseases. Anti-protein aggregating activity of curcumin has been reported earlier. In this study we present that treatment of COS-7 cells expressing mutant rhodopsin with curcumin results in dissociation of mutant protein aggregates and decreases endoplasmic reticulum stress. Furthermore we demonstrate that administration of curcumin to P23H-rhodopsin transgenic rats improves retinal morphology, physiology, gene expression and localization of rhodopsin. Our findings indicate that supplementation of curcumin improves retinal structure and function in P23H-rhodopsin transgenic rats. This data also suggest that curcumin may serve as a potential therapeutic agent in treating RP due to the P23H rhodopsin mutation and perhaps other degenerative diseases caused by protein trafficking defects.

## Introduction

Retinal degenerations are a group of heterogeneous diseases of the retina, which result in irreversible blindness. Age related macular degeneration (AMD) and retinitis pigmentosa (RP) are the most common degenerative diseases of the retina. AMD is characterized by loss of central vision whereas RP is typically characterized as a rod-cone dystrophy in which the genetic defects cause cell death of rod and cone photoreceptors, predominantly the degeneration of rod photoreceptors [1], [2], [3]. RP affects around 1.5 million people worldwide. This disease can be inherited in autosomal dominant, autosomal recessive, X-linked or syndromic forms [4]. Mutations in more than 40 genes are associated with RP [5 RetNet web site–: Available: http://www.sph.uth.tmc.edu/Retnet/disease.htm Accessed 2011 May 25]. Mutations in rhodopsin are the most common cause of RP [6], [7]. Rhodopsin is a G-protein coupled receptor and the most abundant protein in rod photoreceptor cells. It consists of the polypeptide opsin and a single covalently bound molecule of the chromophore, 11-*cis* retinal. Many mutations have been found in the rhodopsin gene and these are distributed along the intradiscal, transmembrane and the cytoplasmic domains of the protein. Mutations in rhodopsin have been classified into three different groups based on their phenotype in heterologous cells. Class 1 mutant proteins are expressed at levels similar to the wild type rhodopsin, form the rhodopsin chromophore with 11-cis retinal and are transported to the cell surface. Class-II mutants form aggregates, remain in the endoplasmic reticulum and do not bind 11-cis retinal to form the chromophore. Class III mutants are expressed at low levels, remain in the endoplasmic reticulum (ER) and form rhodopsin chromophore poorly, cause abnormal trafficking of the protein and result in the formation of aggregates which are retained near the endoplasmic reticulum [8], [9], [10]. The P23H missense mutation is a class-III mutant and accounts for the largest fraction of cases in the world due to rhodopsin mutations [6], [7], [11].

Though the genetic link between the P23H mutation in rhodopsin (P23H-R) and RP have been well established, the mechanism underlying photoreceptor degeneration due to this mutation is yet to be understood. Evaluation of heterologous cells expressing P23H-R demonstrated that the mutant rhodopsin is prone to aggregate formation and is retained in the ER through interactions with elements of the ER's quality control chaperone machinery and targeted for subsequent degradation by the ubiquitin-proteasome pathway [12], [13], [14]. Aggregated mutant rhodopsin was shown to accumulate near the centrosome, recruit cytoskeleton proteins and form perinuclear aggresome-like structures [12], [13], [14]. Co-expression of wild type (wt) and P23H-R demonstrated that the mutant rhodopsin interacts with wt protein and forms cytoplasmic inclusions [12], [13], [14]. Similarly, P23H-R mutation in a Xenopus model also showed formation of aggregates in photoreceptors [15], [16], [17]. Mouse and rat models with P23H-R mutation developed photoreceptor degeneration due to defective trafficking of rhodopsin [18], [19], [20]. These studies suggested that a potential dominant negative effect exerted by P23H-R leading to aggregation and mislocalization of rhodopsin might be the underlying cause of photoreceptor cell death in RP [13], [14].

Misfolding of proteins leading to self-aggregation and altered protein trafficking has been implicated in other degenerative diseases such as Alzheimer's Disease. Inhibition of aggregate formation and favoring the re-folding or clearance of improperly folded proteins have been considered as potential strategies to treat these conditions. In the past decade, a large number of synthetic and natural compounds have been evaluated for their therapeutic potential in treating protein trafficking defects [21]. Several substances like molecular chaperones, growth factors, small molecules, retinoids and anti-oxidants are known to protect cells from the toxic events mediated by misfolded and aggregated proteins [22], [23], [24], [25], [26], [27], [28]. Despite these advances, compounds that are potentially effective in treating retinal degenerations due to protein misfolding have not been identified.

In recent times, a significant increase has been noted in the evaluation of beneficial effects of several natural products for their therapeutic potentials. Among these natural products, phytochemicals mainly polyphenols have been studied extensively with the goal of identifying a suitable natural compound with strong therapeutic effect and minimal toxicity. Polyphenols are derived from many components of the human diet, including dark chocolate, peanuts, green and black tea, red wine, olive oil, fruits, vegetables and curry spices [29]. Some of these phytochemicals which are the components of anti-inflammatory and wound healing agents of traditional eastern medicine are being investigated for their therapeutic potential in treating cardiovascular diseases, inflammatory disease, diabetes, cancer etc [30], [31], [32], [33], [34], [35], [36], [37]. One such compound, curcumin (1,7-bis(4-hydroxy-3- methoxyphenyl)1,6-heptadiene-3,5-dione), a principal component of turmeric, is well known for its antitumor, antioxidant, and anti-inflammatory properties [38], [39], [40], [41]. Curcumin is a member of curcuminoid family and proven to inhibit the formation of aggregates and promote the trafficking of accumulated proteins [23], [24]. The polyphenolic structure of curcumin, (two phenol rings connected by α, β-unsaturated carbonyl groups) allows it to cross the blood brain barrier. Anti-protein aggregating activity of curcumin has been studied and established in several neurodegenerative diseases [42], [43],[44],[45].

In order to determine whether curcumin exerts anti-protein aggregating activity in the retina and rescue photoreceptors from degeneration due to misfolded rhodopsin, we tested the effect of curcumin on cell lines and rats expressing the P23H rhodopsin. Our studies demonstrated that administration of curcumin can inhibit the formation of mutant rhodopsin protein aggregates, protect the cells from toxic events associated with protein aggregation, improve retinal morphology and function. These results establish the potential therapeutic use of curcumin in treating retinal degenerations caused by misfolded or aggregated rhodopsin.

## Results

### Disruption of protein aggregates by curcumin

**Figure 1:**
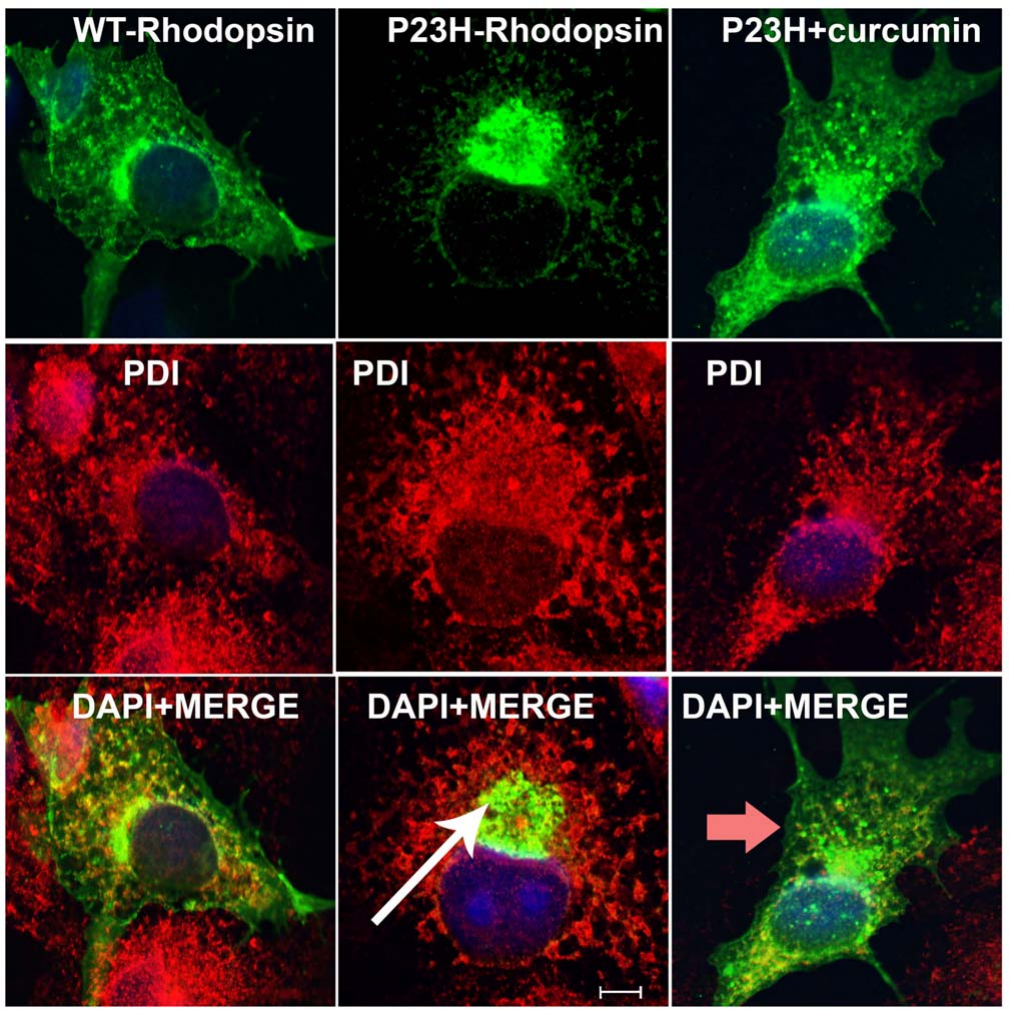
Dissociation of mutant protein aggregates in presence of curcumin. To test the anti-protein aggregating property of curcumin, COS-7 cells were transfected with wt (A–C) and mutant rhodopsin (D–F); supplemented with curcumin (G–I), immunolabeled with anti-V5 (green) and anti-PDI (red) antibodies. Mutant rhodopsin aggregates (white arrow) were dissociated upon treatment with curcumin (red arrow). In presence of curcumin, the expression pattern of mutant rhodopsin is similar to the expression pattern of wt rhodopsin protein. Overlays of images from first two rows on DAPI-stained nuclei of the respective cells (blue) were shown in the bottom panel. Scale bar is 5 µM.

To assess the effect of curcumin on protein aggregation, COS-7 cells expressing wt or mutant (P23H-R) rhodopsin with V5 tag were supplemented with 5 µM concentration of curcumin and analyzed for the presence of protein aggregates by immunocytochemistry followed by fluorescent microscopy. The concentration of curcumin used in this study is determined on the basis of published reports (23). These cells were co-labeled with ER maker- PDI. Consistent with previously published data the wt rhodopsin was found to be localized to the plasma membrane (Fig. 1A) whereas the majority of mutant rhodopsin formed perinuclear aggregates (Fig. 1B) [12], [14]. Treating cells expressing P23H rhodopsin with curcumin resulted in a decrease in the formation of mutant protein aggregates. In these cells, the sub-cellular distribution pattern of P23H rhodopsin was observed to be similar to that of the wt protein (Fig. 1C). Mutant rhodopsin in these cells was found to be predominantly localized to the plasma membrane. In contrast, treatment with curcumin or vehicle had no affect on localization pattern of wild type rhodopsin (data not shown).These data indicate that curcumin treatment may alter the subcellular distribution pattern of mutant rhodopsin.

### Curcumin crosses blood brain and blood retina barrier

**Figure 2:**
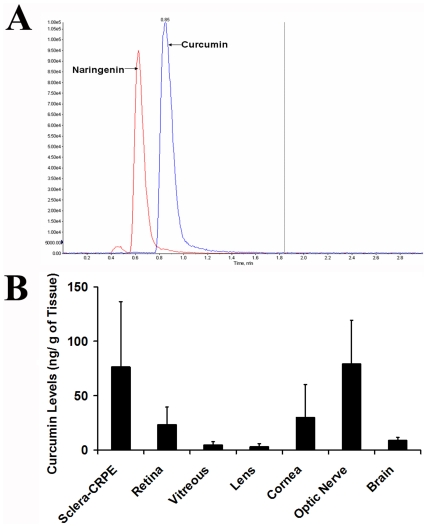
Bioavailability of curcumin. Amount of curcumin detected by LC-MS/MS analysis in the eye and brain tissues of curcumin administered SD rats. (A) Representative LC-MS/MS chromatogram of curcumin (standard) and naringenin (internal standard). (B) Delivery of curcumin to ocular tissues and brain of SD rats following oral administration. Data are expressed as mean ± SD for n = 4.

To investigate the bioavailability of curcumin in brain and ocular tissues, 100 mg/kg body weight of curcumin was administered to control SD rats by gavage and the amount of compound reaching brain and various eye tissues after 2 hours of administration was estimated by LC/MS-MS (Fig. 2A, B). The amount of curcumin administered to rats for these studies was reported to be in the well tolerable doses used in human clinical studies [24], [46]. Evaluation of brain tissue of curcumin administered rats revealed the presence of 0.009 ng of curcumin/ mg tissue weight. Analysis of different eye tissues of rats administered with curcumin detected the presence of curcumin in sclera, retinal pigment epithelium (RPE), retina, optic nerve, cornea, vitreous and lens. The highest amount of curcumin (0.076–0.079 ng/mg tissue) was observed in the Sclera+RPE and optic nerve. In the retina and cornea the amounts of curcumin were found to be around 0.026–0.030 ng /mg of tissue weight. Vitreous and lens showed the presence of lowest amounts of curcumin (0.003–0.004 ng/mg tissues weight). Presence of curcumin in the retina and brain after 2 h of gavage indicates that orally administered curcumin indeed crosses blood-brain and blood-retina barriers.

### Curcumin improves retinal morphology

**Figure 3:**
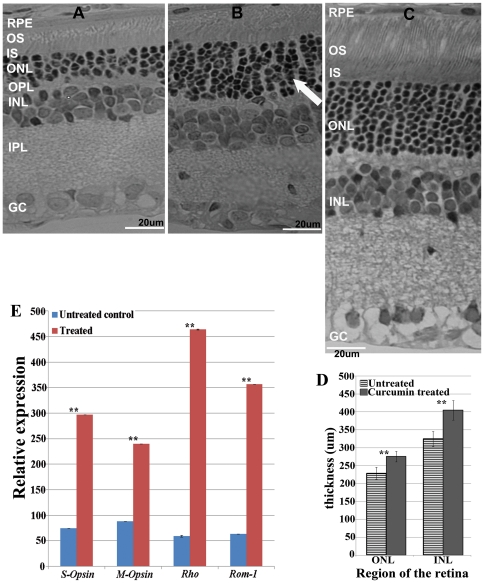
Improved retinal morphology and gene expression of P23H transgenic rats upon administration of curcumin. Light micrographs of retinal sections from untreated control (A) and curcumin treated (B) and wild type Wistar rat retina (C) P23H-R transgenic rats demonstrated a significant improvement in retinal morphology in curcumin administered rats compared to untreated controls. OS = outer segments; IS = inner segments; ONL = outer nuclear layer; OPL = outer plexiform layer and INL = inner nuclear layer; IPL = inner plexiform layer; GC: Ganglion cell layer. The thickness of the ONL and INL in curcumin treated rats were quantified using Aperio Image Scope software Ver. 10.2.2.2319 program (D). We found significant increase in the thickness of both ONL (p≤3.6∧-07) and INL (p≤2∧-05) of the retina of curcumin treated P23H rats when compared to vehicle treated P23H rats. The results are presented as Mean± SD with ** denoting p-values<0.005. Quantitative expression of rod photoreceptor specific markers *Rho and Rom-1* and cone specific makers *S-Opsin* and *M-Opsin*(E) demonstrated an increased expression in curcumin treated rat retinas (Gray bars) is compared to untreated controls (white bars). Gene expression data is calculated from at least 3 independent samples, each of which was analyzed at least in 3 replication reactions. The results are presented as Mean± SD with ** denoting p-values<0.005.

The effect of curcumin on the retinal structure of P23H-R transgenic rats was evaluated by comparing the retinal morphology of treated and untreated transgenic rats (Fig. 3). Consistent with the earlier observations, untreated control P23H transgenic rats developed severe retinal degeneration by postnatal day 60 (P60). The photoreceptor outer and inner segments at this age were observed to be shorter and the outer nuclear layer was found to be thin with 2–3 rows of nuclei. Whereas, transgenic rats administered with curcumin from P30–P60 showed a significant improvement in their retinal morphology by preserving 6 to 7 rows of photoreceptor nuclei (Fig. 3B). The photoreceptor outer segments of these treated rats were observed to be well preserved compared to untreated P23H-R transgenic controls (Fig. 3A) and the wild type control rat retina (Fig. 3C). Improved retinal morphology observed in curcumin administered rats indicates preservation of photoreceptors and the potential beneficial effects of curcumin on retinal degeneration. However no significant difference was observed in the progression of retinal degeneration in vehicle treated rats compared to untreated control rats (data not shown). The thickness of the outer nuclear layer (ONL) and Inner nuclear layer (INL) in curcumin treated and vehicle treated rats was quantified using the Aperio Image Scope software Ver. 10.2.2.2319 program. This analysis revealed a significant increase in the thickness of both ONL (p≤3.6∧-07) and INL (p≤2∧-05) of the retina of curcumin treated P23H-R transgenic rats when compared to vehicle treated P23H-R transgenic rats (Fig. 3D).

### Levels of expression of photoreceptor marker genes is improved upon curcumin treatment

The levels of expression of photoreceptor specific genes in the retina of treated and untreated P23H-R transgenic rats was studied to further evaluate the observations of morphological analysis. Semi quantitative real-time PCR analysis of retinal tissue from curcumin administered P23H-R transgenic rats revealed a significant increase in the expression levels of rod photoreceptor specific markers, rhodopsin and ROM-1) and cone photoreceptor specific markers (S-Opsin and M-Opsin) when compared to their levels in untreated P23H-R transgenic rats indicating preservation of photoreceptor cells (Fig. 3E).

### Curcumin improves retinal physiology

Electroretinography (ERG) was performed on curcumin administered and untreated P23H-R transgenic rats to determine the effect of curcumin treatment from P30 to P70 on retinal physiology in these animals. A significant improvement in retinal function was observed in curcumin treated rats when compared to the untreated after 40 day treatment regimen as measured by the a- and b-wave amplitude of the full field scotopic and the b-wave amplitude of the photopic ERG.

**Figure 4:**
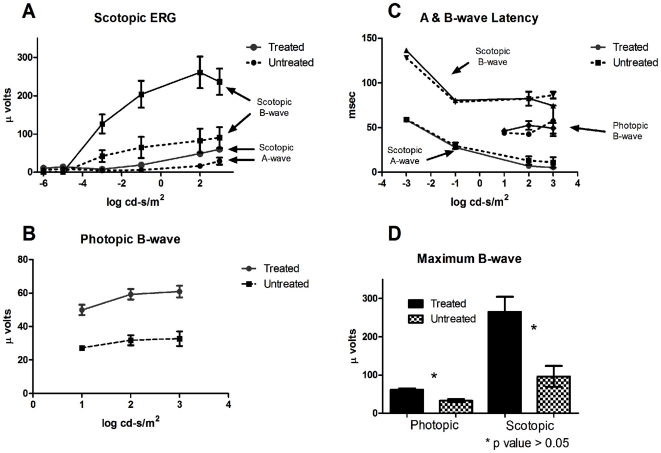
ERG response from untreated Line 1 P23H-R transgenic animals (n = 5, pattern or dashed lines in all panels) and those treated with curcumin at 100 mg/kg body weight dose (n = 13. Solid lines/bars in all panels). (A) The treated animals have improved scotopic A and B-wave amplitudes and improved (B) photopic B-wave amplitudes across all intensities compared to untreated rats. (C) The amplitude latencies remain unchanged in untreated rats suggesting no appreciable change in photoreceptor function. While A and B-wave amplitudes are clearly improved in the treated animals, a statistically significant (students t-test p<0.05) difference is observed in the maximum scotopic and photopic B-wave amplitudes of treated rats when compared to untreated P23H-R transgenic controls (D).

The protection afforded by curcumin treatment was observed across all measurable intensities (Fig. 4A & B). The mean maximum scotopic a-wave amplitude in untreated rats was 28.96 µV (±9.3 µV, n = 5) while rats treated with curcumin had a mean maximum a-wave amplitude of 60.09 µV (±6.8 µV, n = 13) (p = 0.012)The mean maximum scotopic b-wave amplitude in untreated animals was 96.39 µV (±27.1 µV, n = 5) while animals treated with curcumin had a mean maximum scotopic b-wave amplitude of 264.8 µV (±39.7 µV, n = 13) (p = 0. 012) (Fig. 4D). The mean maximum photopic b-wave amplitude in untreated rats was 32.95 µV (±4.15 µV, n = 5) while rats treated with curcumin had a mean maximum photopic b-wave amplitude of 61.3 µV (±3.4 µV, n = 13) (p = 0. 002) (Fig. 4B, D). The implicit time of the ERG amplitudes is unchanged across all intensities between the treated and untreated groups (Fig. 4C). This suggests that the remaining photoreceptors in the retina of treated and untreated animals are functionally similar and untreated animals have either reduced photoreceptors or shortened OS. Both of which are known to be true from previous work by Machida et al [47].

### Curcumin alters the subcellular localization of P23H rhodopsin in cells

**Figure 5:**
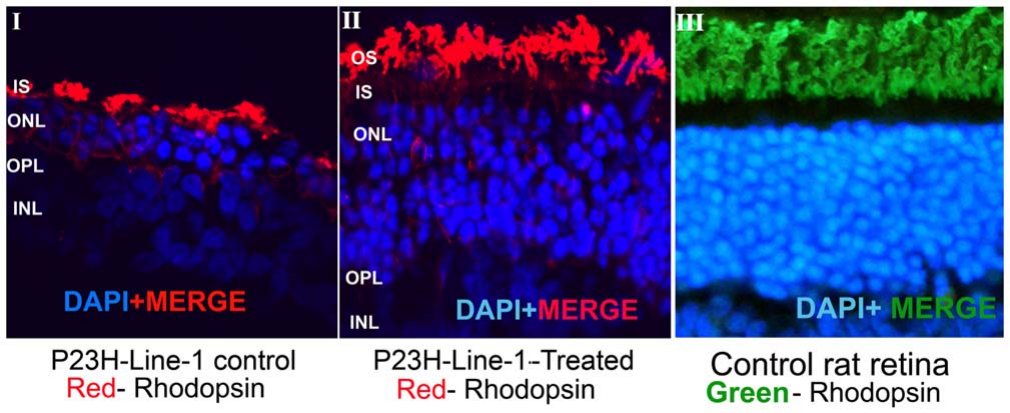
Translocation of Rhodopsin to photoreceptor outer segments in presence of curcumin. Immunohistochemical analysis of retinal sections of curcumin treated P23H-line-1 transgenic rats with rhodopsin demonstrated the translocation of Rhodopsin protein to photoreceptor outer segment region. In control untreated retinal sections rhodopsin was found to be accumulated near the inner segment region of photoreceptors. Red indicates the labeling of rhodopsin in panels I and II and green indicates the rhodopsin labeling in panel III. Nuclei are stained with DAPI. Scale bar is 5 µM. The localization of rhodopsin is observed away from the nuclei in curcumin treated rats contrary to the localization of rhodopsin observed adjacent to the nuclei in untreated control rats thus suggesting the translocation of rhodopsin to outer segments.

To investigate the effect of curcumin on localization of rhodopsin protein in the retina, cryo-sections of curcumin administered and untreated control P23H-R transgenic rat retina were labeled with anti-rhodopsin antibody. The rhodopsin signal in untreated P23H-R transgenic rats was localized adjacent to the remaining few rows of nuclei in the ONL (Fig. 5- I). In contrast, the rhodopsin localization was observed in the outer segment region in the retinas of curcumin administered P23H-R transgenic rats (Fig. 5-II). The pattern of localization of rhodopsin in these retinas is consistent with the localization observed in the retinas of wild type rats (Fig. 5-III). These results indicate that administration of curcumin substantially alters the localization of rhodopsin. It is likely that curcumin facilitates the translocation of rhodopsin to outer segment region or preserves the outer segment structure or both.

### Curcumin decreases endoplasmic reticulum (ER) stress in cells

**Figure 6:**
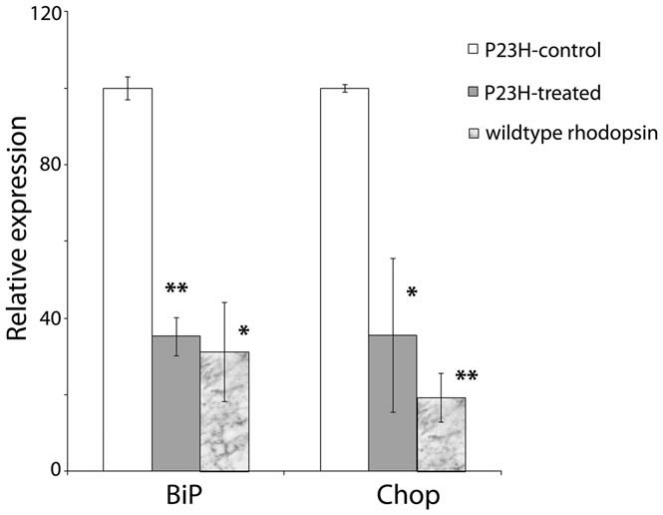
Decreased expression of ER stress markers in curcumin treated COS-7 cells expressing mutant rhodopsin. Quantitative expression of ER stress responsive genes *Grp78/Bip* and *Chop* were determined by qRT-PCR. Expression values were presented on an arbitrary scale (*y*-axis) after normalization with the house keeping gene RPL-19. Data represent the mean (±SD) on an arbitrary scale and were calculated from at least three independent observations. P<0.001 (**), and P<0.025(*).

Previous studies reported that the P23H rhodopsin induces ER stress and activates unfolded protein response (UPR) [14], [48]. To investigate the effect of curcumin on ER stress, the levels of expression of two genes robustly induced by ER stress, *Grp78/Bip* and *CHOP*, were measured in cells expressing wt and P23H rhodopsin in presence and absence of curcumin. Quantitative real time PCR analysis of curcumin supplemented mutant rhodopsin expressing cells demonstrated a significant decrease in the expression of *Grp78/BiP* and *CHOP* compared to their levels in untreated cells (P = 0.001) (Fig. 6). Supplementation of curcumin to cells expressing wt protein did not cause significant alteration in the level of expression of ER stress marker genes when compared to their levels in untreated cells.

**Figure 7:**
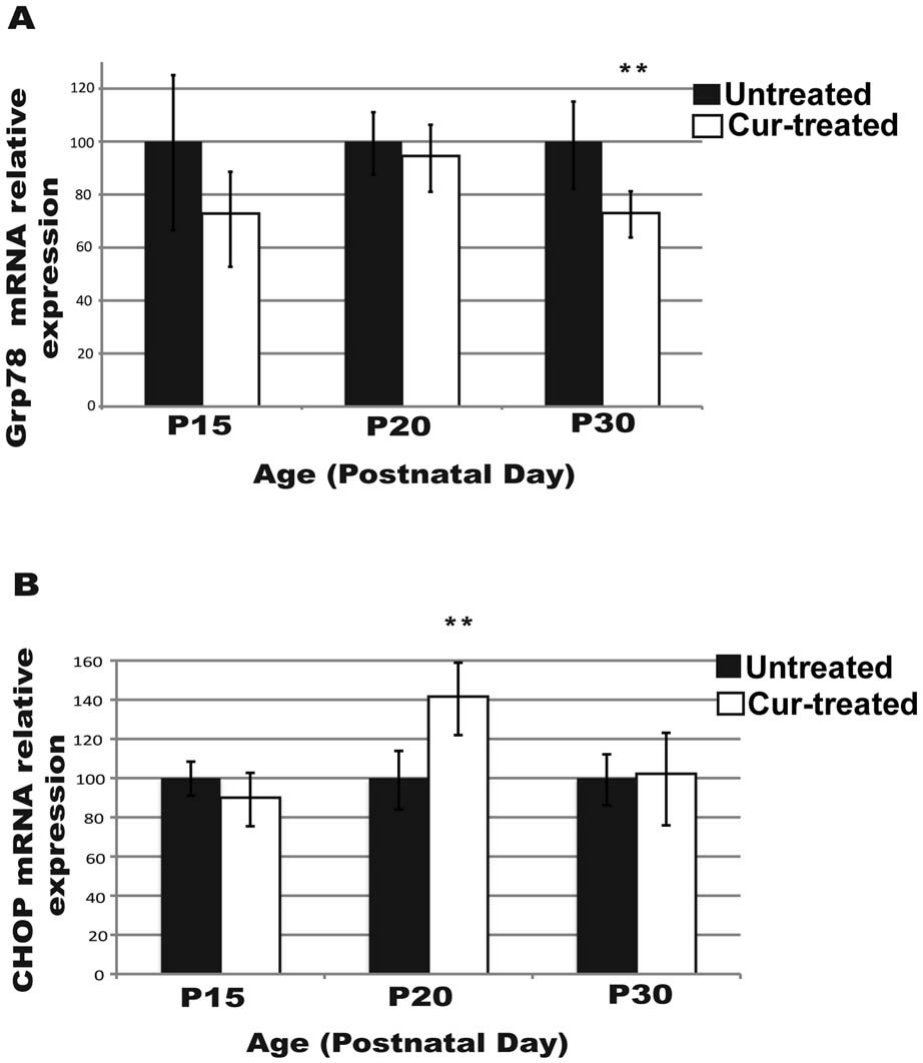
Modulation of ER stress response in curcumin treated P23H-R retina. Expression of ER stress response genes (*A*) *Grp78/BiP* and (*B*) *CHOP* in the retina of P23H-R transgenic rats treated daily with (100 mg/kg body weight) curcumin from postnatal day seven (P7) to P15 or P20 or P30 and age matched untreated controls . Expression values are presented on an arbitrary scale after normalization with the house keeping gene *RPL-19*. Data represents the mean (± SD) expression levels in curcumin treated animals (white) relative to untreated controls (black) at each time point with ** denoting p-values<0.01.

Effect of curcumin on ER stress in the retina of P23H-R transgenic rats was also studied by comparing the expression levels of *Grp78/BiP* and *CHOP* in the retinas of P23H-R transgenic rats treated with curcumin from P15 to P30 and untreated controls. Rats treated with curcumin showed higher levels of *Grp78*/*BiP and Chop* mRNA at P20 compared to their levels at P15 and P30. Comparison of the expression profile of these genes between the treated and untreated groups revealed that the levels of *Grp78/Bip* decreased gradually and the expression was significantly lower in treated rats by the end of treatment on P30. Although the levels of expression of *CHOP* increased significantly at P20 in curcumin treated rats, subsequently they decreased significantly by P30. Additional analysis is needed to determine if prolonged treatment of curcumin will result in further reduction in the levels of *CHOP* (fig. 7).

The studies on cells transfected with P23H-rhodopsin indicated that curcumin treatment may reduce mutant rhodopsin aggregation and therefore allieviate ER stress. However, the studies on P23H-R transgenic rats require further analysis to determine the affect of curcumin treatment on ER stress *in vivo*.

## Discussion

The present study investigated the therapeutic potential of curcumin in treating retinal degenerations due to misfolded rhodopsin. These results demonstrate that curcumin a) dissociates the mutant protein aggregation; b) significantly improves the retinal morphology and physiology of heterozygous P23H-R transgenic rats; c) facilitates the translocation of mutant rhodopsin to appropriate cellular compartment in cell culture and *in vivo*; and d) decreases ER stress in transiently transfected cells expressing P23H rhodopsin.

A wide variety of therapeutic strategies to treat retinal degenerations are being pursued actively [26], [49]. Easy access of retinal tissue to directly deliver therapeutic material makes these studies feasible. Gene replacement therapy by delivering missing genes to the retina by way of injection has been shown to be effective in treating a recessive retinal degeneration lebers congenital amaurosis due to mutations in the RPE65 gene [50], [51]. Several other potential therapies for retinal dystrophies are currently under development or evaluation [52], [53], [54], [55]. Despite these advances, designing therapies for retinal degenerations caused by dominant negative mutations is still a major challenge as the strategy should include removal of the mutant protein in addition to supplementing the missing functional protein. Most of the gene based therapies are specific to diseases due to mutations in particular genes or even specific only to certain types of mutations in a gene. There are more than 200 genes associated with retinal conditions with a large number of mutations in some of these genes (RetNet web site-: Available: http://www.retina-international.org, accessed 2011 June 6; Human Gene Mutation Database:- Available: http://www.hgmd.cf.ac.uk/ac/index.php, accessed 2011 June 6). The number of patients with RD due to any specific mutation is limited. Therefore, therapeutic strategies that are applicable to treat larger number of patients are more effective.

Mutations leading to altered conformation of proteins have been shown to be the underlying cause of several dominant neurodegenerative diseases including dominant retinitis pigmentosa. Protein misfolding leading to altered trafficking and aggregation are common features of these conditions [14]. In general, normal synthesis of proteins is followed by their folding into three-dimensional conformation. Misfolded and unfolded ER client proteins can lead to ER stress and increased cell death through a number of mechanisms including, disruption of important cellular processes such as protein trafficking and degradation. Protein misfolding in the ER initiates natural cellular defense known as the Unfolded Protein Response (UPR) which leads to the up-regulation of multiple target genes, such as Grp78/*BiP* and *Chop*. However, due to persistent accumulation of mutant proteins, the ability of cellular defense against protein folding decreases dramatically. In diseases like RP multiple factors including protein misfolding, altered trafficking, induction of ER stress and reduced proteasomal activity play a significant role in the pathogenesis. These interlinked pathways are predicted to have multiple potential sites for therapeutic intervention. Therefore identification of suitable pharmacological agents targeting the mutant protein, activation of chaperones which in turn facilitate protein folding and enhance the translocation of proteins from ER is beneficial.

Small molecules like curcumin which are known to have multiple therapeutic properties could be effective in treating diseases caused by protein aggregation. Curcumin has been widely used as a coloring agent in food as well as a traditional Indian “Ayurvedic" medicine. Because of its use as a food additive, potential for cancer chemoprevention and decrease in amyloid plaque formation curcumin has undergone toxicological screening and pre-clinical investigation in various disease models and found to be safe [46], [56], [57]. In addition, curcumin is also reported to reduce oxidative damage and amyloid pathology [43], [58]. Recently, direct interaction of curcumin with amyloid β (Aβ), α-synuclein and transthyretin and inhibition of their aggregation has been demonstrated [59]. Until now the exact mechanism of action of curcumin in treating degenerative diseases or various cancers is not understood completely. Several studies demonstrated that curcumin exerts its anti-oxidant and anti-carcinogenic activity by altering the activity of NF-kB, AKT/mTOR, AP-1, NRF2, and protein kinases [60]. Curcumin was also reported to be a more potent free radical scavenger than other antioxidants like vitamin E [61]. Since many pathological states of the degenerative diseases especially neurodegenerations including retinal degeneration involve aggregation of proteins, oxidative stress, and chronic inflammatory response, curcumin has been considered a promising compound to treat degenerative diseases caused by such factors. Recent reports demonstrated beneficiary effects of curcumin against a light induced damage model, chronic anterior uvetis, and cataract formation [62], [63], [64], [65], [66]. These studies also indicated that curcumin exerts its protective effect by modifying the activity of NF-kB, AKT, AP-1, NRF2.

Dissociation of P23H-R aggregates, improvement of retinal morphology and physiology observed upon curcumin treatment in our studies in addition to its reported antioxidant, anti-inflammatory and anti-amyloid plaque formation properties support the potential of curcumin as a therapeutic agent to treat degenerative diseases caused by mutated and misfolded proteins. Although the precise mechanism underlying the photoreceptor rescue in P23H-R transgenic rats by curcumin is unclear, the results of our studies suggest that curcumin may exert its effects through modulation of ER stress and UPR pathways. These observations further suggest that treatment with curcumin may relieve the toxic events associated with mutant rhodopsin protein aggregates. These studies also indicate that modulation of ER stress is likely, but one of several mechanisms by which curcumin enhances photoreceptor cell survival. Since photoreceptor degeneration in RP is associated with induction of UPR and protein aggregation, minimizing or dissociating protein aggregates and thereby reducing ER stress may be of great therapeutic benefit in prolonging the survival of photoreceptors. Further studies are required to understand the exact molecular mechanism underlying the anti-aggregation properties of curcumin.

The evidence provided in this study indicates the therapeutic potential of curcumin in treating RP due to P23H-R mutation. Although the 100 mg/kg body weight dose of curcumin used in study was reported to be clinically safe, additional careful evaluation is needed to establish the duration of preservation of photoreceptors upon treatment with curcumin, length of preservation after termination of the treatment, minimal effective dose and toxicity. Curcumin is likely to be effective in delaying the onset, slow the progression of RP due to P23H rhodopsin mutation and other degenerations due to protein trafficking defects. Administration of curcumin in combination with other therapies such as gene replacement therapy may be more effective in treating diseases due to dominant negative mutations.

## Methods

Reagents and antibodies used in this study: Mouse monoclonal anti-rhodopsin antibody (1 in 1000 dilution- chemicon, Temecula,CA), anti-mouse Alexa fluor 555 (1 in 5000 dilution, Invitrogen, Carlsbad, CA), Rabbit anti-PDI antibody (1 in 200 dilution, Abcam, Cambridge, MA). Anti-V5 antibody (Invitrogen, Carlsbad, CA), Anti-Rabbit Alexa fluor 488 (1 in 1000 dilution, Invitrogen, Carlsbad, CA), curcumin (Sigma- Aldrich, St.Louis, MO, USA). Super Script™ First-Strand Synthesis System (Invitrogen, Carlsbad, CA, USA).

### DNA construct and site directed mutagenesis

The pcDNA 3.1 n V5 DEST rhodopsin (Wt) construct was generated by Gateway cloning strategy (Invitrogen corp, Carlsbad, CA). As per the manufacturer's instructions the pENTER- directional-TOPO entry clones were created by PCR amplification of the cDNA sequence corresponding to the entire coding region of human rhodopsin. These entry clones were used for recombination reaction in the Gateway pcDNA 3.1 nV5 DEST vector carrying the V5 tag. The P23H-rhodopsin construct was prepared by using the PCR- mediated site directed mutagenesis kit (Invitrogen corp, Carlsbad, CA). By PCR amplification and direct sequencing of the entire clone, all the above constructs were confirmed to be in-frame cloning with the fusion tag.

### Animal Maintenance

Homozygous P23H rhodopsin-line-1 transgenic rats were provided by Dr. Matthew LaVail (University of California at San Francisco School of Medicine). Animals used for the present study are heterozygous P23H-line-1 transgenic rats, produced by breeding P23H-line-1 homozygous rats with age matched control Sprague Dawley rats (SD). These studies were conducted in accordance with the ARVO Statement for the Use of Animals in Ophthalmic and Vision Research, and with approval of the Committee on the Use and Care of Animals of the University of California San Diego.

### Preparation of curcumin

To test the *in vitro* effect of curcumin, stocks were prepared by dissolving curcumin in DMSO. To study the effect of curcumin on P23H aggregate formation, 5 µM concentration of curcumin was added to cells along with the transfection complex and incubated at 37°C. After 48 h of transfection, cells were washed and processed for immunolabeling. For administrating curcumin to P23H line-1 transgenic rats, curcumin was dissolved in alimentum (hypoallergic milk powder). To study the effect of curcumin on the morphology and physiology of P23H line 1 transgenic rat retina, 100 mg/ kg body weight of curcumin dissolved in alimentum was administered everyday by oral gavage from P30-till P70 days. For evaluation of gene expression, curcumin was administered from postnatal day 7 (P7) to P30.

### Cell culture and Immunofluorescence analysis of transfected COS-7 cells

Culturing of COS-7 cells and Immunofluorescence analysis of post transfected cells was carried out as described earlier [67], [68]. Immunofluorescence was visualized with a Zeiss laser scanning confocal microscope. Images were captured using appropriate filters and lasers.

### Quantitative real time PCR

Isolation of total RNA from COS-7 cells transfected with rho constructs, curcumin administered and untreated control rat retina, cDNA synthesis and quantitative real-time PCR were performed as described previously[68], [69]. Comparative Ct method was used to calculate the expression levels of ER specific markers as described earlier [48], [70], [71].

### Bioavailability of curcumin

Bioavailability studies were carried out in control Sprague Dawley (SD) rats by administering 100 mg/ kg body weight curcumin orally. Animals were sacrificed after 2 h of administration; brain and eyes were collected and snap frozen. Eyes were dissected in frozen condition to isolate the various ocular tissues. Curcumin was extracted from rat eye and brain tissues by acetonitrile protein precipitation method using naringenin as an internal standard. The calibration curves for analysis of curcumin was prepared only in rat brain tissue in the concentration range of 1–500 ng/ml using 100 ng/ml naringenin as internal standard. Sample analysis was carried out using an API-3000 triple quadruple mass spectrometry (Applied Biosystems, Foster City, CA, USA) coupled with a PerkinElmer series-200 liquid chromatography (Perkin Elmer, Walthm, Massachusetts, USA). Analytes were separated on Kromasil C18 column (2.1×50 mm, 5 µm) using 5 mM ammonium formate in water pH 4.0 (A) and acetonitrile (B) as mobile phase (30∶70 (A∶B) v/v) with flow rate of 0.25 ml /min and total run time of 3 min. Curcumin and naringenin (internal standard) were analyzed in negative ionization mode with following multiple reaction monitoring (MRM) transitions: 367→271 (curcumin); 271→151 (naringenin).

### Histological evaluation

For evaluation of retinal histology, eyes from 5 curcumin administered P23H-line-1 animals and control animals were collected and processed for histology as reported earlier [71], [72]. Tissue sections were stained with H&E and viewed under Nikon light microscope.

### Electroretinograms

Electroretinograms (ERG) were recorded from one eye of the anesthetized P23H line-1 transgenic rat to determine retinal function. Twenty-four hours prior to recording ERG, animals were weighed and allowed to dark adapt overnight (food and water are available ad libitum). All subsequent procedures were performed in the dark with a dark-room safelight or night vision goggles as reported previously [47].

### Immunohistochemistry

For evaluation of localization of rhodopsin in curcumin administered P23H-line-1 transgenic and age matched control rat retina, immunohistochemistry was performed using anti-rhodopsin antibody as described earlier [72], [73].
